# Young People’s Preferences for Family Planning Service Providers in Rural Malawi: A Discrete Choice Experiment

**DOI:** 10.1371/journal.pone.0143287

**Published:** 2015-12-02

**Authors:** Christine Michaels-Igbokwe, Fern Terris-Prestholt, Mylene Lagarde, Effie Chipeta, the Integra Initiative, John Cairns

**Affiliations:** 1 London School of Hygiene and Tropical Medicine, Faculty of Public Health and Policy, Department of Global Health and Development, London, United Kingdom; 2 London School of Hygiene and Tropical Medicine, Faculty of Public Health and Policy, Department of Health Services Research and Policy, London, United Kingdom; 3 University of Malawi, College of Medicine, Blantyre, Malawi; Tulane University School of Public Health, UNITED STATES

## Abstract

**Objective:**

To quantify the impact of service provider characteristics on young people’s choice of family planning (FP) service provider in rural Malawi in order to identify strategies for increasing access and uptake of FP among youth.

**Methods and Findings:**

*A* discrete choice experiment was developed to assess the relative impact of service characteristics on preferences for FP service providers among young people (aged 15–24). Four alternative providers were included (government facility, private facility, outreach and community based distribution of FP) and described by six attributes (the distance between participants’ home and the service delivery point, frequency of service delivery, waiting time at the facility, service providers’ attitude, availability of FP commodities and price). A random parameters logit model was used to estimate preferences for service providers and the likely uptake of services following the expansion of outreach and community based distribution (CBDA) services. In the choice experiment young people were twice as likely to choose a friendly provider (government service odds ratio [OR] = 2.45, p<0.01; private service OR = 1.99, p<0.01; CBDA OR = 1.88, p<0.01) and more than two to three times more likely to choose a provider with an adequate supply of FP commodities (government service OR = 2.48, p<0.01; private service OR = 2.33, p<0.01; CBDA = 3.85, p<0.01). Uptake of community based services was greater than facility based services across a variety of simulated service scenarios indicating that such services may be an effective means of expanding access for youth in rural areas and an important tool for increasing service uptake among youth.

**Conclusions:**

Ensuring that services are acceptable to young people may require additional training for service providers in order to ensure that all providers are friendly and non-judgemental when dealing with younger clients and to ensure that supplies are consistently available.

## Introduction

In sub-Saharan Africa uptake of sexual and reproductive health (SRH) services among youth aged 15–24 remains low, placing millions of young people at risk of poor reproductive health outcomes [1]. High adolescent birth rates (120 per 1,000 girls aged 15–19) place young girls in sub-Saharan Africa at increased risk [1]. In low- and middle-income settings complications related to pregnancy and childbirth represent a leading cause of mortality among adolescent girls [1]. Sexually active young people are also at risk of sexually transmitted infections, including HIV. Young women (age 15–24) are disproportionately affected by HIV with prevalence rates more than twice as high as among men of the same age [2].

Increasing the utilisation of SRH services by young people is therefore critical to improving health outcomes. Recognising the need to make significant improvements in provision of SRH services in Africa, the African Union adopted the Maputo Plan of Action (MPoA): a policy framework for operationalising strategies to achieve health related MDGs. The plan of action outlines six key strategies for improving SRH service delivery including integration of SRH and HIV services, recognition of FP as an essential part of the Millennium Development Goals (MDGs), addressing unsafe abortion, promotion of safe motherhood services and addressing the SRH needs of youth as a key component of the overall policy framework [3]. A signatory to the MPoA, the government of Malawi has intensified efforts to expand access to FP services, with a particular focus on increasing uptake of SRH services by youth [4].

Interventions aimed at increasing utilisation of SRH services by young people have included school- and community-based educational programs, mass-media campaigns, peer education and provision of youth-friendly SRH services in clinical and outreach settings [5]. In Malawi, the expansion of youth-friendly SRH services has received positive reviews [6], yet there has been no rigorous evaluation of the approach to date. In other settings, evidence linking youth focused interventions and increased uptake of SRH services is mixed [7–10], indicating a need for a broader understanding of the determinants of demand for SRH services among this population.

This paper focuses on young people’s preferences for family planning (FP) services in rural Malawi. To date research exploring youth preferences for different types of FP service providers and the relative importance of different elements of service delivery has been limited and has focused on facility based delivery [11]. Where a discussion of community based services has been included, youth may have limited knowledge or experience of this model of service delivery [12, 13]. Understanding what motivates youth to choose between different types of FP service providers, could help identify strategies to make services more accessible and attractive to both current and future users. This study uses a discrete choice experiment (DCE) approach to explore young people’s preferences for formal FP service provision.

DCEs are a quantitative stated preference method which can be used to understand how consumers value different goods or services, called alternatives, as described by a set of attributes. In health economics, DCEs have been used to examine patient preferences for a variety of services and service delivery models [14]. Respondents are presented with a series of hypothetical choices and asked to indicate which of two or more alternatives they would be most likely to choose; this may include an option to choose none. This approach is particularly useful when information on actual choices is unavailable, when there is little variation among currently available alternatives and for exploring preferences for new services not yet available in the market place. DCE data can also be used to develop strategies for the introduction of new services, service delivery models or policies [15–17].

The advantages of DCEs over the analysis of service utilisation data in this context are twofold. Firstly, preferences for new modes of service delivery not currently available in the marketplace can be assessed. This is particularly important given that the aim of the research is to inform the expansion of services into areas with limited or no outreach and community based services. Secondly, service utilisation data can only provide insight into preferences among service users and necessarily excludes the preferences of non-users. This approach would therefore be of limited utility in terms of understanding service delivery factors that may be increasing uptake among individuals who have not previously accessed services. A DCE conducted amongst both current FP users and non-users can be used to identify preferences across both groups.

The objectives of this study were to identify service characteristics that may influence young people’s choice of FP service provider, to understand the relative importance of service characteristics and to model the potential changes in service uptake as services are expanded and improved.

## Methods

### Study Setting

Recent estimates from the Malawi Demographic and Health Survey show that young people aged 15–24 have high levels of unmet need for reproductive health services and are at risk of adverse health outcomes across a variety of indicators [18]. Estimates of contraceptive prevalence in Malawi indicate that 25–27% of married women and 8–21% of unmarried women aged 15–24 report unmet need for reproductive health and FP services [18]. Young women are both more likely to have been tested for HIV and received the result (62.6%) than young men (41.8%) and more likely to be HIV positive. Among young women aged 15–19 and 20–24, HIV prevalence is 4.2% and 6.4% respectively. Among young men in the same age groups HIV prevalence is 1.8% and 2.3%, respectively [18].

In line with national efforts to increase utilisation of SRH services by young people, the Family Planning Association of Malawi (FPAM), a local non-governmental organisation providing SRH services in Malawi, is working towards expanding its outreach and community based service delivery programme. As services are expanded information is required on how to design services to attract young people and effectively increase uptake. In partnership with FPAM, seven communities in one Traditional Authority in Ntcheu District were randomly selected and invited to participate in this research. One of the research communities is a FPAM outreach service delivery site and the remaining six have no formal outreach services or permanent health facilities. At the time of the survey health surveillance assistants and peer educators were operating in selected communities distributing condoms and offering counselling on SRH issues.

### Design of the DCE Survey

The DCE design was informed by a scoping literature review and series of qualitative interviews aiming to identify the main sources of FP in the area and explore factors influencing choice of service provider. These consisted of 12 focus group discussions (FGDs) with a random sample of 15–24 year olds identified through household listing and three key informant interviews with peer educators working in the research communities. Written informed consent was obtained for all participants involved in the qualitative work. For participants 15–17 written informed consent was first obtained from the parent or guardian and then from the participant. Details of the DCE development [19] and results of the qualitative work [20] are summarised briefly here and reported in detail elsewhere.

In the qualitative interviews young people identified a range of service providers including government clinics, private clinics, outreach services and community health workers. Respondents commonly referred to government services as ‘free’ services and private services as ‘paying’ services’. The price of services at the facility level was an important driver of choice for many respondents.

*‘We do go to some other hospitals because of lack of money for example we go to [Health Centre 2] because of lack of money but when you have money one goes to [Health Centre 1]*. *We go to [Health Centre 2] because it’s free of charge*.*’*—Female, 15–19

Distance to facility was viewed as a barrier to access and choice of family planning service provider was weight against both the cost of transport and cost of services at the facility level.

*‘It is possible that the private hospital may be close but because of money problem one may choose to go for free services though it is far away*.*’*—Male, 15–19

Service availability was described both in terms of operating hours and the frequency with which outreach services were offered. When considering accessing outreach services, respondents considered the timing of outreach and the urgency of their needs. This was true for both SRH and non-SRH services.

*‘I can go to [Health Centre 1] because that [outreach] only comes here sometimes only once in a month*. *That means it can be difficult for us to wait for them*. *So it’s better to go to [Health Centre 1] where they work every day*, *if they found you HIV positive they counsel you properly how you can take care of yourself*.*’—Key Informant*

In selecting a health facility, youth described both positive and negative interactions with service providers, though the degree to which this was a factor influencing choice varied.

*‘The doctors*….*aah the female doctors are pompous when addressing us fellow girls*. *That’s why I can’t go to [Health Centre 1] because of the way they behaved at times*.*’*—Female, 15–19

*‘Like at [Health Centre 1] the health workers there they welcome you warmly and we chat with them freely for this reason you are able to be open with them and explain your problem*. *We are able to access the services that we want compared to other health workers*, *so this makes us feel happy*.*’*—Male, 15–19

*‘We are afraid to go to the hospital because when you go there the medical personnel start to ask why you so young wants to start family planning … but despite all this I can still go…’*—Key Informant

Stock outs of commodities and difficulties accessing sufficient family planning commodities presented a challenge for young people and respondents indicated that this also impacted their choice of provider.

*‘[Hospital 1] is the best for family planning services because you can all family planning methods are available than at [Health Centre 2] you might only find condoms no injection or pills and this is same with [Health Centre 1]*. *Then I prefer [Hospital 1]*.’—Male 20–24

*‘As our area is far from these service providers we can sometimes leave here and upon arriving there we won’t find condoms*. *But sometimes they can only give us 3 condoms*, *so we think for how long are we going to use these 3 condoms regarding distance where we are coming from which is far*.*’*—Male, 15–19

The amount of time spent waiting to see a provider at the facility was also identified as a factor influencing choice. Some respondents described this as a function of the number of other services provided at the facility and whether service were provided free of charge; where services are provided for free many young people anticipated that more clients would be present and that this would increase the waiting time. An alternative viewpoint around waiting time was that service providers did not begin work on time or took extended breaks and that this increased the amount of time one could expect to spend waiting.

‘*Waiting time too will be another factor to consider*. *For example*, *at [Hospital 1] as there are many services and at same time it is for free one may find more clients than [Clinic 1] therefore it is better to go for [Clinic 1] where waiting time is not longer*.*’*—Male, 20–24

Other issues related to the discussion around alternative providers raised in the qualitative work included the structure and presentation of the health facility, cleanliness and hygiene standards; however, these were not described as having an impact on respondents’ choice of facility and so were not included as attributes in the DCE. Concerns about confidentiality were highlighted, however as this was linked to both distance (where youth reported travelling further from home to access services in a location where no one would be likely to know who they are) and service provider attitudes, this was not selected as an attribute for this particular DCE.

The final DCE included four labelled alternatives and an opt-out. Labelled alternatives were: government facility, private facility, outreach service and community based distribution agents (CBDAs). Attributes used to describe the alternatives included the distance between the participant’s home and the facility, service delivery frequency, availability of FP commodities, service provider attitudes, waiting time and price. A second unlabelled DCE containing 12 choice tasks eliciting respondent preferences for the configuration of outreach services specifically was also included in the questionnaire. These results are presented elsewhere [21].

**Fig 1:**
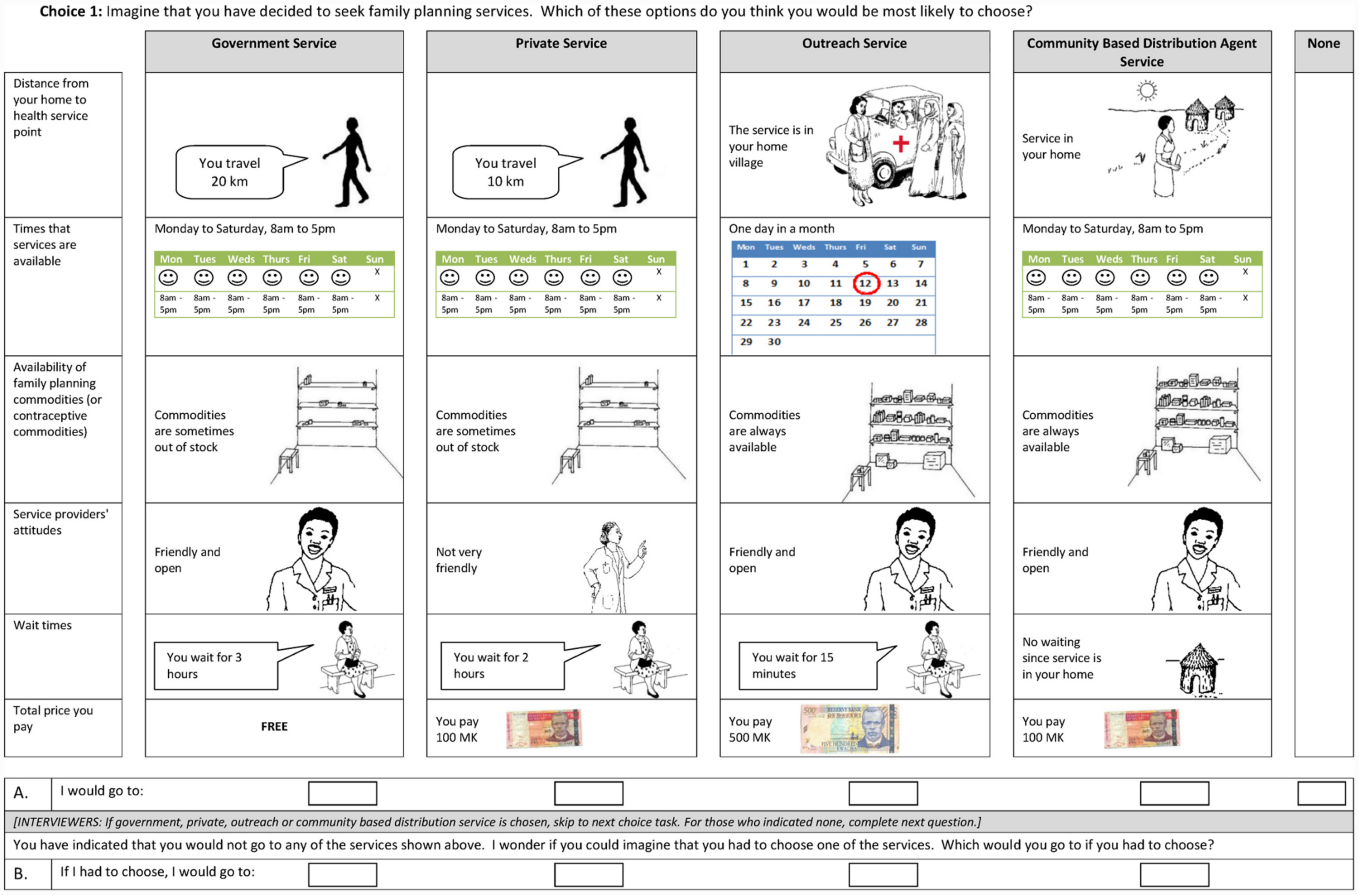
Sample Choice Task of Family Planning Service Provider.

The final experimental design included 4 attributes with 2 levels and 2 attributes with 4 levels (2^13^ x 4^2^). Each of the attributes and levels were alternative specific and four alternative specific constants were included, resulting in 19 coefficients to be estimated. Table 1 presents the final attributes and levels. A full factorial design of this nature would produce 131,072 choice profiles. Given the large number of possible choice profiles, a fractional factorial design was used in order to limit the number of choice sets that each respondent was required to complete. For the same reason, the experimental was specified to include main effects only. The final experimental design was generated using Ngene [22] using a D-efficient design with zero priors (d-error = 0.012). The correlation matrix for the final design is provided in an online appendix (S1 Table). In order to ensure that the final design could be blocked, 20 choice tasks were created. These were divided into four blocks of five choices, meaning that each respondent was presented with five choice tasks related to the choice of FP provider (in addition to the 12 choice tasks relating specifically to the design of outreach services). A sample choice task is provided in Fig 1. Each choice task included an unforced choice between the four service alternatives and the opt-out followed by a forced choice in the event that respondents opted out. The data presented in this analysis relate only to the unforced choice. The pictorial representation of all attributes and levels can be found in an online appendix (S2 Table).

**Table 1:** List of final attributes and levels included in the discrete choice experiment eliciting young people's preferences for FP service providers in Malawi, 2012.

	Government Facility		Private Facility		Outreach Service^a^		Community Based Distribution Agent (CBDA)	
	Description	Code	Description	Code	Description	Code	Description	Code
1. Distance	• 20 km 35 km	20 35	• 10 km 20 km	10 20	• *In your village*		• *In your home*	
2. Service delivery frequency	• *Services available from Monday to Saturday*, *8am to 5pm* ^b^		• *Services available from Monday to Saturday*, *8am to 5pm*		• One day per month	2	• *Services available from Monday to Saturday*, *8am to 5pm*	
					• Once day every other month	1		
3. Availability of FP commodities	• Some FP methods may not be available all the time	-1	• Some FP methods may not be available all the time	-1	• *All FP methods are available*		• Some FP methods may not be available all the time	-1
	• All FP methods are available	1	• All FP methods are available	1			• All FP methods are available	1
4. Service provider attitude	• Service provider is open and friendly	1	• Service provider is open and friendly	1	• *Service provider is open and friendly*		• Service provider is open and friendly	1
	• Service provider is stern and may be rude and scold youth asking for FP	-1	• Service provider is stern and may be rude and scold youth asking for FP	-1			• Service provider is stern and may be rude and scold youth asking for FP	-1
5. Waiting time at the facility	• 1 hour 3 hours	60 180	• 2 hours 30 minutes	120 30	• 15 minutes 45 minutes	15 45	• *No wait*	
6. Price in Malawi Kwacha (MK)^c^	• *Free*		• 100 MK 250 MK	100 250	• Free 50 MK 250 MK 500 MK	0 50 250 500	• Free 50 MK 100 MK 250 MK	0 50 100 250
Additional information about FP methods offered by each provider but not included in the DCE as an attributes	Condoms, oral contraceptives, implants, injectables, intrauterine device (IUD), sterilization		Condoms, oral contraceptives, implants, injectables, IUD, sterilization		Condoms, oral contraceptives, implants, injectables, IUD		Condoms and oral contraceptives^d^	

^a^ Several of the levels for outreach services were fixed to reflect the structure of services delivered by FPAM,

^b^ Attributes in *italics* were fixed to the specified level in order to match alternative descriptions with services available in research communities and anticipated structure of new services,

^c^ 50 Malawi Kwacha was equal to approximately USD$0.20 at the time of the survey in May-June 2012,

^d^ Currently available community distribution includes only condoms. Oral contraceptives were included since plans to expand this service include the distribution of oral contraceptives using trained CBDAs

A pilot was conducted with 40 respondents aged 15–24 to test the DCE choice tasks. Images representing each of the attributes and levels were included in each choice task and revised following pilot participants’ feedback to ensure that they conveyed the intended meaning. An updated experimental design was generated following the pilot reflecting changes in the number of levels and the addition of a community based distribution agent alternative, which had not been identified at the pilot stage. The addition of another choice alternative meant that prior parameter estimates derived from analysis of the pilot data could not be used to inform the final experimental design. Therefore, an efficient design with zero priors was used to generate the final design and the design with the lowest d-error of 145,000 designs evaluated was selected. A design with a low d-error minimises both the variance and covariance between design parameters and allows for the smallest possible standard errors in the final analysis [23]. This allows for allow for more precise estimates of mean parameter values.

Prior to the start of the choice tasks, interviewers reviewed the images used to describe the attribute levels with each respondent, providing examples and a verbal description to ensure correct interpretation of the DCE choices. Choice tasks were framed by asking respondents to imagine that they had already decided to use FP services, and then indicate which of the alternatives they would be most likely to choose, or whether they would choose none. This ensured the preferences of both FP users and non-users were considered. Further discussion around the motivation behind this particular framing has been published separately [19].

The DCE was embedded within a larger questionnaire which included questions related to respondent and household characteristics, knowledge of FP methods, current use of FP and previous use of FP services. The wording and ordering of background questions were also tested in the pilot and refined prior to the survey. Ethical approval was obtained from the Research Ethics Committees at the University of Malawi, College of Medicine and the London School of Hygiene and Tropical Medicine. Witten informed consent was obtained for each participant. For participants aged 15–17 written consent was first obtained from the parent or guardian and then from the participant prior to beginning the questionnaire.

Household listing identified 910 youth between the ages of 15 and 24 in the research communities. Out of these, 620 individuals were randomly selected and invited to participate. Due to the eight month time lag between the initial community mapping and formative research and the administration of the final survey a number of respondents had moved out of the area and we were unable to trace all respondents that were randomly selected; however, of those individuals that could be traced, none declined to participate. The final sample included 540 completed questionnaires.

### Framework for Analysis

Methods for the analysis of choice data are rooted in random utility theory which assumes that consumer choices are probabilistic rather than deterministic. As such individual utility (or satisfaction) can be expressed as:

$$U_{i}=\;V_{i}+\;\varepsilon_{i}$$

Where *U*_*i*_ is the utility obtained from choosing alternative *i* of the alternatives *j* = 1,…, *i*,…,*J* available in a given choice set, C. *V*_*i*_ represents the observable components of choice and *ε*_*i*_ (often called an error term) is a collection of unobserved influences representing heterogeneity in tastes across individuals or errors in measurement or model specification [24]. The generalised notation for *V*_*i*_ is written as:

$$V_{i}=\;\beta_{n}X_{ni}\;+\;\beta_{z}Z_{ni}\;+\;\varepsilon_{ni}$$

Each of the *β* terms represents the weight that individual *n* places on the corresponding variables where *X*_*ni*_ represents design attributes and *Z*_*ni*_ represents individual socio demographic characteristics (SDCs) [24].

Within this framework, the most popular models for the analysis of choice data are the multinomial logit (MNL) and RPL models. In recent years the RPL model has overtaken the MNL in popularity as it offers increased flexibility in accounting for unobserved heterogeneity in preferences. This flexibility is a result of the decomposition of the error component into two parts, a random element *η*_*ni*_ which follows a distribution specified by the analyst, and *ε*_*i*_ which is assumed to be independently and identically distributed. The element *η*_*ni*_ is critical in modelling heterogeneity in respondent preferences since it is correlated over choice sets and is allowed to vary across respondents.

All parameters in the base model and final analysis were alternative specific. The alternative specific constants (ASCs) were included in N-1 alternatives to capture respondent preferences for each alternative relative to alternative without an ASC, which was the government service in this case. Categorical design attributes were effects coded to avoid confounding with the mean (codes indicated in Table 1). Design attributes were specified as random with a normal distribution with the exception of the price parameter which was specified to have a constrained triangular distribution with a lower bound of 0, and the ASCs, which were fixed. Models were estimated using maximum likelihood simulation with 500 Halton draws used in RPL specifications.

In the first stage of analysis a base MNL model was estimated using only design attributes as explanatory variables (these results are provided in the online appendix, S3 Table). In the second stage an RPL model was estimated using only design attributes as explanatory variables. The final RPL model includes interactions between design attributes (i.e. the service characteristics) and SDCs in order to investigate how preferences may vary according to observed individual characteristics. SDCs include age category (15–19 and 20–24 years of age), gender, school attendance and current use of a modern method of FP. Age category and gender were included as interaction terms in order to identify systematic variations in preferences that may point to a need for different programmatic approaches to attracting younger (aged 15–19 years) or older youth (aged 20–24 years) or male and female respondents. Similarly, school attendance was included in order to identify whether young people attending school had different preferences around attending a facility outside of their community compared to a community based service. Current use of FP was included as an interaction term to identify whether respondents who are current service users had systematically different preferences relative to those who are not using family planning. The interaction terms included in the final model were identified using an iterative step wise approach whereby all possible interactions were estimated and those that were not statistically significant removed in subsequent analyses until only interactions that were consistently statistically significant remained. Additional covariates included in exploratory analysis included relationship status and employment status but these were not consistently statistically significant and so were not included in the final model.

Model fit was assessed using both a log likelihood ratio (LLR) test and Aikaike’s Information Criteron (AIC). The results of the final model were used to conduct a series of simulations to assess the impact of changes in service delivery on the uptake of services offered by different providers.

## Results

A summary of respondent characteristics is provided in Table 2. The results of the base model are presented in Table 3 and the final model estimates are presented in Table 4. Results of the LLR test indicate that the final RPL model with interactions provides a better fit than the RPL model which included only service characteristics. This is confirmed by the lower value of the AIC in the final model.

**Table 2:** Study population characteristics in discrete choice experiment eliciting young people's preferences for FP service providers in Malawi, 2012.

		Value	
Variable Name	Description	No. (n)*	%
Younger	Age 15–19	328 (540)	61%
Older	Age 20–24	212 (540)	39%
Female		269 (540)	50%
Male		271 (540)	50%
School	Currently attending school	266 (530)	54%
Relationship	Currently in a relationship	346 (540)	64%
Employed	Has worked in the last 12 months	280 (519)	54%
Sex	Sexually active in the past 12 months	302 (389)	78%
Condom	Used a condom at last sex	216 (389)	56%
FP Use	Used a modern method of FP in the last 12 months	274 (540)	51%
Future FP	Non-users who intend to use FP in the future	245 (269)	91%

**Table 3:** Base comparison RPL model results for discrete choice experiment eliciting young people's preferences for FP service providers in Malawi, 2012.

	Base RPL Model			
Choice	Coefficient	SE^a^	StdD^a^ ^,^ ^b^	SE^a^ ^,^ ^b^
Random Parameters				
*Government*				
Distance	-0.03 ***	0.01	0.04 ***	0.01
FP commodities	1.04 ***	0.08	0.13	0.22
Service providers	0.88 ***	0.08	0.43 **	0.18
Wait time	0.002	0.001	0.01 ***	0.002
*Private*				
Distance	-0.01	0.01	0.01	0.01
FP commodities	0.97 ***	0.12	0.73 ***	0.19
Service providers	0.69 ***	0.10	0.14	0.22
Wait time	-0.01 **	0.004	0.01 ***	0.002
Price	-0.01 ***	0.002	0.01 ***	0.003
*Outreach*				
Frequency	0.18	0.11	0.65 ***	0.09
Wait time	0.003	0.004	0.01 *	0.01
Price	0.003 ***	0.004	0.003 ***	0.0003
*CBDA*				
FP commodities	1.15 ***	0.07	0.60 ***	0.13
Service providers	0.95 ***	0.07	0.06 ***	0.24
Price	-0.005 ***	0.001	0.005 ***	0.001
Nonrandom Parameters				
Private ASC^c^	0.16	0.45		
Outreach ASC	1.71 ***	0.36		
CBDA ASC	0.17	0.31		
None ASC	-1.74 ***	0.31		
**Model Fit Statistics**				
Number of individuals	540			
Number of observations	2700			
Log Likelihood Function	-2913.78			
AIC	5889			

^**a**^ SE = Standard Error, StdD = Standard Deviation,

^b^ Only for random parameters,

^c^ ASC = Alternative Specific Constant.

***p<0.01;

**p<0.05;

*p<0.1.

Likelihood Ratio Test Between Base RPL and Base MNL Model: LL_RPL-MNL_ = 269.88χ^2^_112.0.0.001_(32.9)

**Table 4:** RPL results for discrete choice experiment eliciting young people's preferences for FP service providers in Malawi, 2012.

Choice	Coefficient	SE^a^	StdD^a^ ^,^ ^b^	SE^a^ ^,^ ^b^	Odds Ratio	95% Confidence Interval
Random Parameters						
*Government*						
Distance	-0.021 **	0.010	0.044 ***	0.006	0.98	0.96–1.00
FP commodities	0.908 ***	0.102	0.354 **	0.180	2.48	2.03–3.03
Service provider attitude	0.897 ***	0.084	0.483 ***	0.150	2.45	2.08–2.89
Wait time	0.003 *	0.002	0.005 **	0.002	1.00	1.00–1.01
Private						
Distance	-0.008	0.010	0.011	0.016	0.99	0.97–10.1
FP commodities	0.846 ***	0.160	0.731 ***	0.214	2.33	1.70–3.19
Service provider attitude	0.687 ***	0.103	0.493 **	0.203	1.99	1.62–2.43
Wait time	-0.014 **	0.005	0.017 ***	0.004	0.99	0.98–1.00
Price	-0.005 ***	0.002	0.005 ***	0.002	0.99	0.99–1.00
Outreach						
Frequency	-0.161	0.115	0.564 ***	0.081	0.85	0.68–1.07
Wait time	-0.008 *	0.004	0.021 ***	0.005	0.99	0.98–1.00
Price	-0.003 ***	0.000	0.003 ***	0.000	0.997	0.997–0.998
CBDA						
FP commodities	1.348 ***	0.145	0.488 ***	0.153	3.85	2.90–5.11
Service provider attitude	0.630 ***	0.134	0.241	0.153	1.88	1.44–2.44
Price	-0.009 ***	0.002	0.009 ***	0.002	0.991	0.989–0.994
Non Random Parameters						
Private ASC^c^	-0.321	0.469			0.73	0.29–1.82
Outreach ASC	1.523 ***	0.365			4.59	2.24–9.38
CBDA ASC	-0.008	0.317			0.99	0.53–1.85
None ASC	-1.844 ***	0.316			0.16	0.09–0.29
**Interaction Terms**						
*Government*						
Distance: Age 20–24	0.034 ***	0.008			0.97	0.95–0.98
FP Commodities: In School	0.335 **	0.146			1.40	1.05–1.86
Wait Time: In School	-0.003 **	0.001			0.997	.0994–0.999
*Private*						
FP Commodities: FP User	0.370 *	0.206			1.45	0.97–2.17
*Outreach*						
Wait Time: In School	0.011 **	0.005			1.01	1.00–1.02
*CBDA*						
FP Commodities: Female	-0.541 ***	0.143			0.58	0.44–0.77
FP Commodities: In School	0.356 **	0.148			1.42	1.07–1.91
Svc Prov. Attitude: Age 20–24	0.262 *	0.148			1.30	0.97–1.74
Svc Prov. Attitude: In School	0.506 ***	0.151			1.66	1.23–2.23
Price: FP User	0.004 ***	0.001			1.004	1.001–1.006
Price: In School	0.004 ***	0.001			1.004	1.001–1.006
**Model Fit Statistics**						
Number of individuals	540					
Number of observations	2700					
Log Likelihood Function	-2824					
AIC	5732					

^a^ SE = Standard Error, StdD = Standard Deviation,

^b^ Only for random parameters,

^c^ ASC = Alternative Specific Constant.

***p<0.01;

**p<0.05;

*p<0.1.

Likelihood Ratio Test Between Final Model and Base Model: LR_Final-Base_ = 179.9χ^2^_11.0.0.001_(24.7)

All of the coefficients have the expected sign. The ASCs for both outreach services and the ‘None’ alternative are statistically significant. Overall, the significance of these terms show that respondents positively value outreach services and prefer to access services compared to no services (or any other type of service not listed).

For all types of provider, respondents are significantly more likely to choose a service with friendly and non-judgmental service providers. The strength of preference was broadly similar across the three alternatives that included this attribute with respondents being twice as likely to prefer a service with a friendly provider compared to one with a provider who may be unfriendly or judgemental (government service Odds Ratio (OR) = 2.45, p<0.01; private service OR = 1.99, p<0.01; and CBDA OR = 1.88, p<0.01). Respondents also positively valued having a reliable supply of FP commodities. The strength of this preference varied across provider types and was the strongest for the CBDA provider, with respondents being more than three times more likely to choose this type of provider when a reliable supply of FP commodities was available compared to a provider where the availability of supplies was limited or uncertain (OR = 3.85, p<0.01). Respondents were more than twice as likely to choose a government service (OR = 2.48, p<0.01) or a private service (OR = 2.33, p<0.01) with a reliable supply of commodities.

Distance was negative for both the government and private alternatives; however, the coefficient was only statistically significant for the government facility and the odds ratio of 0.98 (p<0.05) suggests that respondents were not less likely to choose a government facility that was further away. The price coefficient is negative for all three services where a fee may be charged (government services are always free) confirming that youth prefer a service with a lower price. As with distance, the odds ratios for price variables are only slightly below one. However, this represents the change in the odds of choosing a service given a one unit change in price as expressed in Malawi Kwacha (MK) and the minimum price presented was 50 MK (at the time of the survey in May-June 2012, 50 MK was equal to approximately USD$0.20).

In general, waiting time is not a strongly significant influence on the choice of provider for any of the alternatives (government service OR = 1.003, p<0.1; private service OR = 0.99, p<0.05; and outreach service OR = 0.99, p<0.05). The frequency of outreach service delivery does not significantly influence preferences for outreach services (within the range of values presented in the DCE); however, the significance of the standard deviation on this parameter estimate suggests that while the mean value is not significant, there is substantial variation in preferences for this attribute.

Across all alternatives, respondents were 4.59 times more likely to choose an outreach service for FP services over a government, private or CBDA service (OR = 4.59, p<0.01). However, given that the attributes for provider attitudes and FP commodities were fixed as friendly and available respectively, preferences for these attributes are also captured in the ASC for this alternative and cannot be estimated separately. This is therefore likely to be an overestimate of the preference for the outreach services.

The interaction terms reveal that respondent preferences for some service attributes varied according to SDCs. Individuals who were attending school are more likely to choose a government or CBDA service with a reliable supply of FP commodities relative to respondents who were not in school (government service OR = 1.40, p<0.05; CBDA OR = 1.42, p<0.05) and a CBDA service with a friendly service provider (OR = 1.66, p<0.01). Respondents who were in school were slightly more sensitive to wait times for government services (OR = 0.997, p<0.01) but less sensitive to waiting times for outreach services (OR = 1.01, p<0.05), indicating that out of school respondents were more likely to prefer a government service with a shorter waiting time compared to in school respondents and may be willing to wait longer for an outreach service than out of school respondents. School attendees were also less sensitive to the price of FP services delivered by CBDAs (OR = 1.004, p<0.01), though the value of the interaction coefficient (0.004, p<0.01) relative to the mean (-0.009, p<0.01) indicates that the overall direction of preference for price is still negative meaning that respondents still prefer a lower price.

Respondents currently using FP were more likely to choose a private service with a reliable supply of FP commodities compared to respondents not using FP (OR = 1.45, p<0.1) and were less sensitive to the price of FP services delivered by a CBDA (OR = 1.004, p<0.01), though they still preferred a lower price overall. Older respondents (aged 20–24) were slightly less likely to choose a government service that was further away compared to younger respondents (aged 15–19) (OR = 0.97, p<0.01). and were more likely to choose CBDA services if the provider is friendly and non-judgemental (OR = 1.30, p<0.05). Female respondents were almost half as likely (OR = 0.58, p<0.015) to choose a CBDA with a reliable stock of FP commodities compared to their male counterparts, meaning that this feature was a more important consideration for males than females.

A series of simulated scenarios were created to investigate the combined impact of changes in service attributes on the uptake of services by provider (Table 5) compared with the base uptake in the model. The base choice shares from the DCE closely matched the reported utilization for all but the CBDA alternative for which the DCE choice share was greater than reported usage. In all but the first three scenarios, which focused on changes in price alone, simulations indicated that respondents would be likely to move from government and private facility based services toward community based services, if available. Scenarios one through 12 showed an increase in the proportion of respondents that would choose CBDA services and scenarios 13 and 14 were the only ones to show an increased use of outreach services.

**Table 5:** Simulated uptake of different service providers under selected service scenarios, 2012.

**Scenarios** ^a^ ^,^ ^b^ ^,^ ^c^ ^,^ ^d^ ^,^ ^e^ ^,^ ^f^		**Government**	**Private**	**Outreach**	**CBDA**	**Other/None** **
** **		*Total uptake (%)*	*Total uptake (%)*	*Total uptake (%)*	*Total uptake (%)*	*Total uptake (%)*
Reported utilization (unweighted)*		15.7%	4.6%	30.6%	10.2%	3.5%
Reported utilization (weighted)***		24.4%	7.2%	47.3%	15.8%	5.4%
**Base uptake in choice experiment**		**21.2%**	**8.1%**	**43.4%**	**23.4%**	**3.8%**
		*Change (%)*	*Change (%)*	*Change (%)*	*Change (%)*	*Change (%)*
1	All services are free	3.7%	4.2%	-23.8%	19.1%	-3.1%
2	Outreach and CBDA cost 50MK ^ⱡ^, Private services cost 150MK	7.8%	-0.3%	-22.4%	17.9%	-2.9%
3	Outreach and CBDA are free, Private cost 150MK	5.1%	-1.1%	-22.5%	21.7%	-3.1%
4	Outreach, CBDA and Private have friendly providers	-14.0%	6.9%	-18.9%	28.9%	-2.9%
5	Outreach, CBDA and Private have FP commodities in stock	-15.5%	7.1%	-18.5%	29.8%	-2.9%
6	Outreach and CBDA have friendly providers	-13.2%	-2.7%	-15.9%	34.6%	-2.8%
7	Outreach and CBDA have FP commodities in stock	-14.6%	-5.1%	-14.7%	37.1%	-2.7%
8	Outreach and CBDA have friendly providers and FP commodities in stock	-19.8%	-7.2%	-12.1%	41.7%	-2.6%
9	Outreach, CBDA and Private have friendly providers, Private cost 150MK, Outreach cost 50MK	-13.3%	0.9%	-19.1%	34.2%	-2.8%
10	Outreach, CBDA and Private have FP commodities in stock, Private cost 150MK, CBDA cost 50MK	-14.2%	1.7%	-13.2%	28.4%	-2.6%
11	Outreach, CBDA and Private have friendly providers and FP commodities in stock, Private cost 150MK, CBDA cost 50MK	-19.7%	2.3%	-11.2%	31.2%	-2.6%
12	Outreach, CBDA and Private have friendly providers and FP commodities in stock, Private cost 150MK, Outreach cost 50MK	-19.9%	1.5%	-17.2%	38.2%	-2.7%
13	Only outreach has friendly providers and FP commodities in stock, Private cost 150MK, CBDA cost 50MK	-17.0%	-6.7%	43.8%	-20.5%	0.4%
14	Only outreach has friendly providers and FP commodities in stock, Private cost 150MK, CBDA cost 50MK, waiting times for Government and Private are doubled and distance is 35km	-15.0%	-6.3%	41.6%	-20.6%	0.3%

* Reported utilization was obtained from survey data asking respondents where they had accessed FP services in the last 12 months.

** In the case of reported utilization, ‘Other’ indicates reported utilization of provider types not included in the DCE. In the simulations, ‘None’ indicates an opt-out response.

*** In the full sample only 65% of respondents had accessed FP services in the past 12 months. Unweighted proportions reflect the choices of the full sample. Weighted proportions reflect the market shares for the individuals that have accessed services in the past 12 months.

^a^ For all simulations, the distance for Government and Private services was fixed at 20km, and 0km for Outreach and CBDA.

^b^ For all simulations Government, Private and CBDA services were assumed to be available 6 days a week from 8am to 5pm. Outreach services are available one day per month.

^c^ For all simulations providers are assumed to be friendly and FP commodities readily available unless otherwise specified.

^d^ For all simulations wait times are set to 2 hours for Government, 1 hours for private and 30 minutes for Outreach unless otherwise specified.

^e^ For all simulations prices are set to 0 (free) unless otherwise specified.

^f^ All comparisons are against the base uptake in the choice experiment.

^ⱡ^ MK = Malawi Kwacha. 50 Malawi Kwacha was equal to approximately USD$0.20 at the time of the survey in May-June 2012

## Discussion

The results highlight the importance of provider attitudes and the availability of FP commodities on choice of provider. This confirms the findings of previous work [25–27]. The impact of the availability of FP commodities was highlighted in qualitative work related to this study [20] but has not previously been explored in the literature. Distance was not as strong a predictor of choice as was anticipated based on responses in the qualitative work and the literature review [20, 28–30]. However, only the government and private alternatives were outside the research communities and these were less preferred in general than the community based services.

Given the significance of the ASC for the outreach alternative in the RPL analysis, we would expect this alternative to be favoured over the CBDA alternative in the simulation scenarios. However, the simulations show that CBDA services were consistently preferred to other service providers across a variety of scenarios. This could be a result of a lack of variation in the attributes used in the experimental design; FP commodities were available in outreach for all choice sets and the service providers were always presented as being friendly and non-judgemental. This means that in addition to reflecting the value that respondents placed on the outreach service in and of itself, the ASC also includes the value that respondents placed on these two attributes. (In a labelled design, the ASC is used to understand respondent preferences for attributes in relation to the label. Since preferences can only be measured for the attributes included in the experiment, all other unobserved influences are captured in the ASC. For example, respondents may make inferences about an alternative based on their perceptions of the label or interpret it as a proxy for omitted attributes.) This may have led to an overestimation of the value placed on outreach services relative to other service alternatives. The simulation exercise therefore provides additional insight into the probability that respondents are likely to choose a particular provider.

Differences between predictions from the DCE and reported use of FP services are presented in. While it is not possible to rule out the usual limitation of stated preference techniques (hypothetical bias), this discrepancy is also likely to stem from differences between the range of service providers included in the DCE survey compared those actually available in the research communities and surrounding area and the methods of FP that they offer. In particular, peer educators and health surveillance workers were classed as CBDA services in the reported use figures. At the time of the survey these individuals were only able to offer condoms whereas in the DCE oral contraceptives were assumed to be available reflecting plans for expanding CBDA services.

In the simulations, CBDA services remained popular across a variety of scenarios, and uptake of outreach services only increased when all other alternatives did not have friendly providers and FP commodities in stock (scenarios 13 and 14). However, under these scenarios more than 80% of youth would be likely to choose an outreach provider suggesting that in cases where all other alternatives are unsuitable, outreach services have the potential to reach a substantial number of youth. Indeed, formative qualitative work suggests that this scenario is not incompatible with the current structure of available services in the research communities [31] indicating that the availability of outreach services could have a substantial impact on service uptake among young people.

An area of concern may be the slight increase in individuals who would be likely to choose the ‘none’ alternative in scenarios 13 and 14. This is in contrast to a decrease in those choosing ‘none’ in all other scenarios, which suggests that as the structure of service delivery changes and individuals switch providers, a small number of individuals are unlikely to switch to outreach services and may choose not to access any service instead. This is an area that warrants further investigation in future work.

The DCE results indicated that the frequency of outreach service delivery was not an important predictor of choice on average, but that there was significant heterogeneity around the mean parameter estimate. This may be because some youth had a hard time imagining waiting a month or two month between service delivery days, or that some would be willing to wait and could plan in advance for a non-urgent service like FP so did not find the frequency to be an issue. This contradicts findings from related qualitative work where participants expressed frustration with outreach service providers coming infrequently or coming to the community only once and then failing to return [20]. The discrepancies between the two studies may stem from the framing of the attribute in the DCE, which did not include an element of uncertainty around the timing of service delivery. Further work may be required to disentangle the influences of frequency and consistency of service availability and service provider attitudes on preferences for outreach services.

Limitations of this work include challenges in making inferences about patterns of switching providers and opting out. The choice tasks were framed by asking youth to imagine that they had already decided to access services. This was helpful in gaining an understanding of the preferences of non-users, but means that it is not possible to predict new uptake among those who have not previously used FP services. Switching behaviour has been examined to some extent through the simulations, but more work could be done particularly to explore uptake among non-users.

## Conclusion

This study has shown that young people are significantly more likely to choose a friendly provider with an adequate supply of FP commodities. Overall, our results are consistent with the view that given the right tools, existing service providers and service delivery models can adequately address youth needs [29]. Improving the quality of community based services shows more potential for expanding youth access to SRH services in rural areas compared with facility based services and may be an important tool for increasing the uptake of SRH services in this population. Ensuring that services are acceptable to young people may require additional training for service providers in order to ensure that all providers are friendly and non-judgemental when dealing with younger clients and to ensure that supplies are consistently available.

## Supporting Information

S1 Table Choice of Family Planning Provider Experimental Design Correlation Matrix. (XLSX)

S2 Table Images for Choice of Family Planning Provider. (PDF)

S3 Table Base Multinomial Logit Results. (PDF)
