# Supplementary material for: Young People’s Preferences for Family Planning Service Providers in Rural Malawi: A Discrete Choice Experiment
Source: PLoS One. 2015 Dec 2;10(12):e0143287. doi: 10.1371/journal.pone.0143287 (PMC4667908; doi:10.1371/journal.pone.0143287)
Supplement: S2 Table — (PDF) [file pone.0143287.s002.pdf]

ILLUSTRATIONS FOR CHOICE OF HEALTH FACILITY

01

One of the characteristics of a health facility is where it is located. In some cases, the place may be far and you may have to walk or get transport to go there. These pictures describe the distance to the facility in terms of the number of kilometers you would need to travel in order to get from home to the health facility.

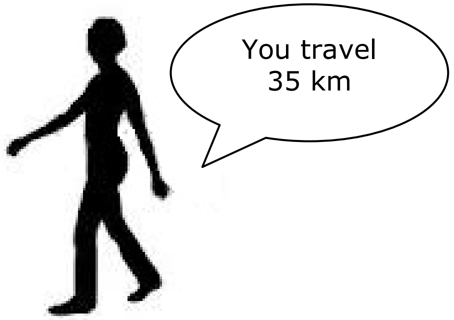

Here you see a picture of a person walking along with the words 'you walk for 35km. This means that if you were to walk from your home to the facility the distance would be 35 km which is about the same as the distance from here to Ntcheu town. You will see a picture like this with different distances, for example, you may have a facility that is 20 km away or 10km away.

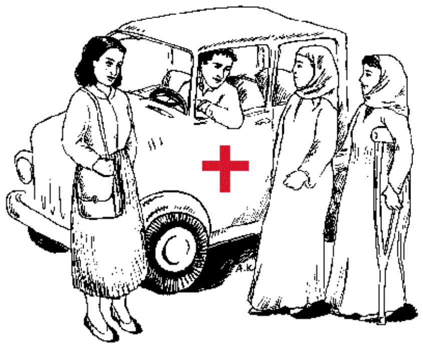

This picture is showing a health worker coming to the village to meet clients. Remember that the health workers are not there all the time, rather they come to the village just for the day to deliver services.

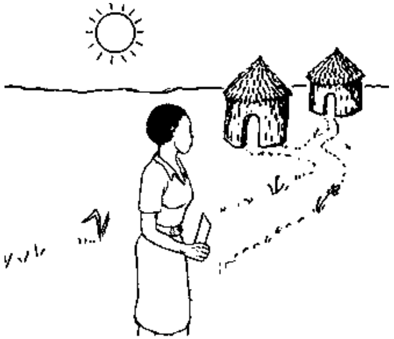

This picture is showing a trained volunteer who can come to your house and provide family planning like condoms or pills in your home. This means that you won't have to travel at all to receive family planning, but the methods will be brought to you.

02

These pictures are showing the total fee that you will pay for receiving the family planning method of your choice. In some cases, the services that we are showing may be available for free and in other cases there may be a fee. Here are two examples of pictures showing how much the service costs.

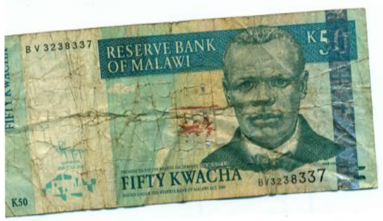

This picture of a 50 Kwacha note indicates that you would be asked to pay 50 Malawi Kwacha at the clinic for both the consultation and the family planning method of your choice.

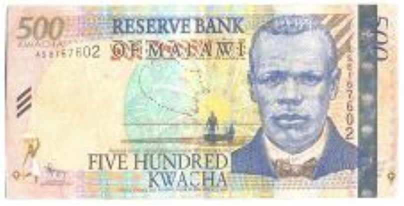

This is a 500 Kwacha note. This means that you would pay 500 Kwacha for the family planning method and consultation

03

These pictures are showing the days and times that the services are available. In some cases like in a government hospital or a private clinic, services will be available from Monday to Saturday. But for outreach services, the service won't be available all the time. For example, outreach services could come one a month or once every two months.

Mon	Tues	Weds	Thurs	Fri	Sat	Sun
☺	☺	☺	☺	☺	☺	X
8am - 5pm	8am - 5pm	8am - 5pm	8am - 5pm	8am - 5pm	8am - 5pm	X

This shows a weekly calendar with happy faces on the days Monday through Saturday. This means that you can access services at this facility on these days. The only day that there are no services is Sunday. On the bottom row we see the times 8am to 5pm. This means that you can get services at the facility between these hours.

Mon	Tues	Weds	Thurs	Fri	Sat	Sun
1	2	3	4	5	6	7
8	9	10	11	12	13	14
15	16	17	18	19	20	21
22	23	24	25	26	27	28
29	30					

This shows a monthly calendar. Here only one day in the month is circled in red. This means that in the whole month, services will only be available one day. This will only be used to describe outreach services that are coming to your village.

04	*These pictures are describing the availability of the family planning commodities.*	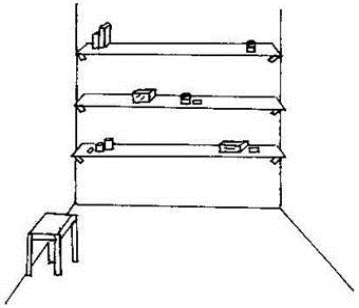<br>*This picture is showing a health facility with few items on the shelves. This doesn't mean that there are never commodities here, but indicates that the family planning method that you would like may not be available, so there is a chance that you may not find what you are looking for.*	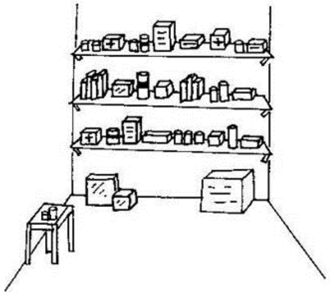<br>*This means that when you go to this facility you will not have a problem obtaining the method of family planning that you want.*
05	*These pictures are describing the attitude of the service provider and how the service provider treats you.*	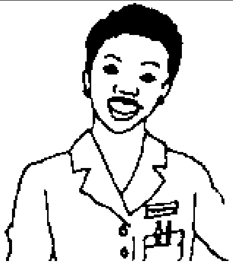<br>*This picture shows a service provider who is smiling and friendly. The aim of this picture is to convey that the service provider at this facility is friendly and open. This picture represents a provider that treats everyone equally and is not judgmental. This type of provider is dedicated to his/her job, and is free to answer questions and provide counseling.*	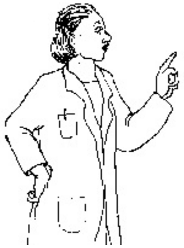<br>*This picture shows a service provider with a stern demeanor. This type of provider may ask many questions and scold youth who ask for family planning. This type of provider may not seem interested in providing a good service; he/she can be rude at times and may rush patients through a consultation without providing adequate counseling according to patient needs.*
06	*These pictures are describing the time that you wait from when you arrive at the health facility to the time that you get to see the service provider for your consultation.*	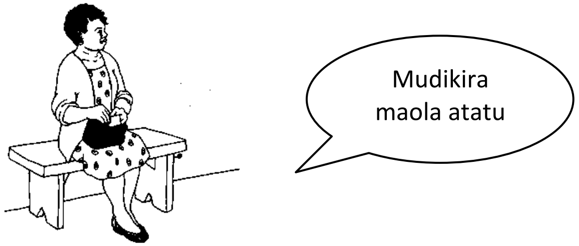<br>*This picture shows a person waiting to see a service provider to receive family planning and the words indicate the length of time. In this example, a client is waiting for three hours.*	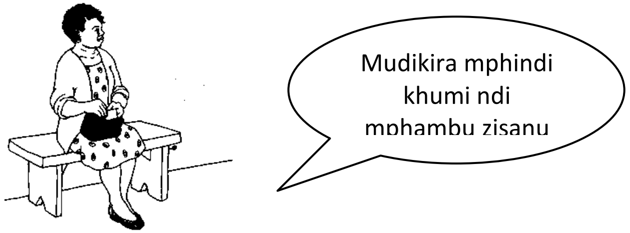<br>*This is a similar example, but in this case, the length of the waiting time is 15 minutes. The main difference is seen in the words so it is important to note the difference when we look at the choice scenarios below.*
