# Supplementary material for: Young People’s Preferences for Family Planning Service Providers in Rural Malawi: A Discrete Choice Experiment
Source: PLoS One. 2015 Dec 2;10(12):e0143287. doi: 10.1371/journal.pone.0143287 (PMC4667908; doi:10.1371/journal.pone.0143287)
Supplement: S3 Table — (PDF) [file pone.0143287.s003.pdf]

This appendix presents the results of the base multinomial logit (MNL) specifications used in the preliminary analysis of the discrete choice experiment assessing young people's preferences for type of family planning service provider.

	Base MNL Model		
**Choice**	**Coefficient**		**SE[a]**
Government			
Distance	-0.017	**	0.007
FP commodities	0.766	***	0.057
Service provider attitude	0.659	***	0.055
Wait time	0.002	*	0.001
Private			
ASC[b]	-0.425		0.347
Distance	-0.012	*	0.007
FP commodities	0.789	***	0.092
Service provider attitude	0.512	***	0.080
Wait time	-0.002		0.002
Price	-0.003	**	0.001
Outreach			
ASC	1.312	***	0.287
Frequency	-0.109		0.087
Wait time	-0.003		0.003
Price	-0.002	***	0.000
CBDA			
ASC	0.063		0.240
FP commodities	1.011	***	0.059
Service provider attitude	0.777	***	0.057
Price	-0.004	***	0.001
None ASC	-1.754	***	0.240
Model Fit Statistics			
Number of individuals	540		
Number of observations	2700		
Log Likelihood Function	-3048.71		
AIC	6135.4		

[a] SE = Standard Error, [b] ASC = Alternative Specific Constant
***p<0.01; **p<0.05; *p<0.1.
